# Myogenic Differential Methylation: Diverse Associations with Chromatin Structure

**DOI:** 10.3390/biology3020426

**Published:** 2014-06-19

**Authors:** Sruti Chandra, Carl Baribault, Michelle Lacey, Melanie Ehrlich

**Affiliations:** 1 Center for Bioinformatics and Genomics, New Orleans, LA 70112, USA; E-Mails: schandr1@tulane.edu (S.C.); ehrlich@tulane.edu (M.E.); 2 Tulane Cancer Center, Tulane University Health Sciences Center, New Orleans, LA 70112, USA; E-Mails: crlbrblt@gmail.com (C.B.); mlacey1@tulane.edu (M.L.); 3 Department of Mathematics, Tulane University, New Orleans, LA 70112, USA; 4 Program in Human Genetics Program, Tulane University, New Orleans, LA 70112, USA

**Keywords:** DNA methylation, histone modification, myoblasts, DNaseI hypersensitivity, differentiation, enhancers, promoters, Polycomb group repression, muscle

## Abstract

Employing a new algorithm for identifying differentially methylated regions (DMRs) from reduced representation bisulfite sequencing profiles, we identified 1972 hypermethylated and 3250 hypomethylated myogenic DMRs in a comparison of myoblasts (Mb) and myotubes (Mt) with 16 types of nonmuscle cell cultures. DMRs co-localized with a variety of chromatin structures, as deduced from ENCODE whole-genome profiles. Myogenic hypomethylation was highly associated with both weak and strong enhancer-type chromatin, while hypermethylation was infrequently associated with enhancer-type chromatin. Both myogenic hypermethylation and hypomethylation often overlapped weak transcription-type chromatin and Polycomb-repressed-type chromatin. For representative genes, we illustrate relationships between DNA methylation, the local chromatin state, DNaseI hypersensitivity, and gene expression. For example, *MARVELD2* exhibited myogenic hypermethylation in transcription-type chromatin that overlapped a silenced promoter in Mb and Mt while *TEAD4* had myogenic hypomethylation in intronic subregions displaying enhancer-type or transcription-type chromatin in these cells. For *LSP1*, alternative promoter usage and active promoter-type chromatin were linked to highly specific myogenic or lymphogenic hypomethylated DMRs. Lastly, despite its myogenesis-associated expression, *TBX15* had multiple hypermethylated myogenic DMRs framing its promoter region. This could help explain why *TBX15* was previously reported to be underexpressed and, unexpectedly, its promoter undermethylated in placentas exhibiting vascular intrauterine growth restriction.

## 1. Introduction

Analysis of genome-wide profiles of DNA methylation (methylomes) in humans is revealing new associations between differentiation and DNA epigenetics [1,2]. Especially informative are studies that combine DNA methylome analysis with whole-genome profiling of chromatin epigenetics, including histone modifications by chromatin immunoprecipitation/next generation sequencing (ChIP-seq) [3,4,5,6,7]. These studies have demonstrated that relationships between DNA methylation and gene expression are much more complex than previously assumed and often highly dependent on the context of the gene region, the surrounding chromatin epigenetics, and the cell type [8,9,10]. For example, the paradigm that more gene methylation is associated with less expression has many exceptions, most notably that more methylation in the body of a gene has been observed to be globally correlated with more gene expression [3,9,11]. 

While increases in gene-body CpG methylation together with increases in histone H3 lysine 36 trimethylation (H3K36me3) are positively associated with transcription in genome-wide studies [12], extensive DNA hypermethylation of CpG-rich promoters and sequences immediately downstream of the transcription start site (TSS) represses transcription [11,13]. Hypomethylation of enhancers is correlated with their activity [14]. Evidence is accumulating that changes in DNA methylation also help direct alternative splicing [15]. Our previous analyses of myogenesis-associated differential DNA methylation and gene expression from whole-genome profiles reinforce the hypothesis that vertebrate DNA methylation plays multiple roles in regulating gene expression including little-studied ones, such as helping to finely tune expression levels, limiting the spread of promoter- or enhancer-type chromatin, and silencing repressive DNA elements [16,17,18].

Our myogenic epigenetic studies have been focused on tissue-specific changes in DNA methylation determined by comparing reduced representation bisulfite sequencing (RRBS) profiles of myogenic progenitor cells (myoblasts, Mb, and myotubes, Mt) and 16 types of cell cultures derived from non-cancerous tissues other than muscle [17]. We found that 10,048 and 9592 CpG sites displayed myogenic hypomethylation or hypermethylation, respectively [18]. Although only ~5% of total CpG sites are identified by RRBS, the site distribution is skewed towards genes and CpG islands (CGIs) and away from highly conserved repetitive elements [2]. RRBS is, therefore, highly informative about gene activity. We also compared skeletal muscle with 14 non-muscle tissue samples and determined that 11,255 CpGs displayed muscle-associated hypomethylation while only 761 sites exhibited muscle-associated hypermethylation. The comparison of myogenic differential methylation at the myogenic progenitor and adult tissue stages indicates that ~97% of myogenic hypermethylated sites are lost at some time after the Mt (fused Mb) stage. This extremely large loss of Mb- and Mt-hypermethylated sites was preferentially from specific subsets of genes associated with early differentiation, including homeobox genes [18]. About 30% of myogenic hypomethylated sites in Mb and Mt were also observed in skeletal muscle. Moreover, ~73% of the muscle-hypomethylated sites were present in muscle but not in the Mb and Mt, indicative of DNA demethylation after the Mt stage. 

In this report, we examine differentially methylated DNA regions (DMRs) instead of DM CpG sites in myogenic *vs*. nonmyogenic cells or tissues. These were determined by our recently developed DMR-identification method optimized for RRBS data [19]. While identifying DM sites has the advantages of allowing the detection of isolated DM CpG sites that might affect binding of sequence-specific transcription factors [20,21,22] and helping minimize underreporting of differential methylation in regions with poor RRBS coverage, profiling DMRs has other important advantages. Tissue-specific DNA methylation often exerts its effects regionally [23,24], not by methylation of a specific protein-binding site in DNA, but rather by strong or moderate overall levels of methylation in a region in one cell type and a low level in another [25,26]. Moreover, when detecting DMRs, the threshold for differential methylation for each covered CpG site can be lower because multiple CpGs are being scored. In addition, comparison of numbers of DM sites gives overrepresentation of long clusters of such sites, while such overrepresentation is minimized when comparing numbers of DMRs. 

Using myogenic DMR profiles and ENCODE data [27] for predicting the type of chromatin in which the DMRs are embedded (e.g., weak promoter, active promoter, strong enhancer, weak enhancer, Polycomb group-repressed) [28], we compare genome-wide myogenic DMRs and chromatin epigenetic states to further reveal the variety of probable functions of differential DNA methylation. We illustrate specific associations between DNA and chromatin epigenetics for four representative genes, *TBX15*, *TEAD4*, *LSP1*, and *MARVELD2*. The first three of these genes are preferentially transcribed in the muscle lineage, and the last one is tightly repressed specifically in myogenic progenitor cells. Analysis of the subgene location, chromatin epigenetics, and expression of these genes suggests important roles in differentiation for the observed myogenic DMRs, including roles that are not just secondary to changes in histone modification.

## 2. Experimental Section

### 2.1. Determination of DMRs

Fifty-seven cell-culture or tissue DNA samples (including technical or biological replicates) from the ENCODE RRBS database used for determination of DMRs and DM sites were previously described [9,18]. They included nine Mb or Mt samples provided by our laboratory and characterized as to their quality by immunocytochemistry. RRBS databases are available ([29], DNA methylation by RRBS, Richard Myers, HudsonAlpha Institute for Biotechnology). Individual DM sites associated with myogenesis were required to have an observed change in the difference in the proportion of methylation (PMD) of at least ±0.5 (50%) at the 0.01 significance level as determined by fitted binomial regression models. For myogenic DMR identification, we used our recently designed novel UPQ algorithm that increases the sensitivity and specificity of comparisons of multiple RRBS datasets by adjusting single-site binomial regression *p*-values for coverage score and sample size [19]. The DMR detection routine incorporates the Uniform Product distribution. R scripts implementing our methods are available [30]. DMRs covering two or more sites were mapped to the RefSeq gene isoform closest to distal or proximal end using the “nearest” function in the R/bioconductor package “rtracklayer” [31]. To reduce the false-positive rate, results were filtered to include only DMRs with a mean difference in PMD of at least plus or minus 0.25 (25%) and a log *p*-value < −10.

### 2.2. Chromatin and Expression Data Sources

Profiles of DNaseI hypersensitivity, chromatin state segmentation, histone modifications, and strand-specific RNA-seq are from ENCODE data [29], namely Open chromatin by DNaseI HS (Gregory Crawford, Duke University [32]); Chromatin state segmentation by HMM [28] and Histone modification by ChIP-seq (Bradley Bernstein, Broad Institute); and Strand-specific RNA-seq (Long RNA-seq, >200 nt poly(A)^+^, Tom Gingeras, Cold Spring Harbor Laboratory). For quantification of RNA-seq data by Cufflinks [33], we used non-strand-specific profiles from ENCODE (Long RNA-seq, >200 nt poly(A)^+^, Barbara Wold, California Institute of Technology) or newly generated RNA-seq data (Mb and Mt RNA-seq libraries prepared from poly(A)^+^ RNA (50-bp paired-end reads, Illumina Hiseq 2000 San Diego, CA, USA; Gregory Crawford, Duke University and Melanie Ehrlich, Tulane University [16]).

## 3. Results and Discussion

### 3.1. Genome-Wide Analysis of Associations between Myogenic Differential Methylation, Chromatin States, and Transcription Start Sites

#### 3.1.1. Determination of Myogenic DMRs

We compared RRBS profiles for our set of Mb and Mt (MbMt) with the analogous profiles for 16 types of nonmuscle cell cultures to determine MbMt DMRs using our UPQ algorithm [19]. Because Mb and Mt had very similar methylation profiles that were very different from those of nonmuscle cells, they were analyzed as a single set to determine MbMt DMRs. We identified 3250 hypomethylated and 1972 hypermethylated myogenic DMRs. The hypermethylated DMRs were significantly longer than the hypomethylated ones (*p* < 10^−16^, non-parametric test) and contained more individual DM CpG sites (*p* < 10^−16^) but the site density was higher for the hypomethylated DMRs (*p* = 0.04). These results suggest more spreading of de novo methylation [23,34,35] than of demethylation. 

#### 3.1.2. Association of Transcription with Myogenic Hypomethylation *vs*. Hypermethylation

To look for an association of myogenic hypomethylated or hypermethylated DMRs with the steady-state levels of RNA from their associated genes, we used previously determined relative expression levels for Mb *vs*. 19 nonmuscle cell cultures (exon-based microarray expression profiles [36]). For this analysis, we considered only DMRs that were within the body of their associated gene or 2 kb from either end (gene ± 2 kb) and had microarray expression data for their nearest gene. This gave a subset of 1708 hypomethylated and 1001 hypermethylated DMRs. The association of hypermethylated DMRs with >2-fold transcription downregulation in Mb was much stronger than that of hypomethylated DMRs (*p* = 0.004, Fisher’s exact test). However, the difference was only modest, 65 hypermethylated MbMt DMRs, 6.5%, *vs*. 68 hypomethylated MbMt DMRs, 4.0%. Upregulation >2-fold in Mb was not significantly associated with hypomethylated *vs*. hypermethylated DMRs (121 hypomethylated DMRs, 7.1%, *vs.* 71 hypermethylated DMRs, 7.1%, *p* = 1). Therefore, changes in DNA methylation in the extended gene region were mostly not associated with gene expression in a simple way. 

#### 3.1.3. Association of Myogenic Hypomethylation *vs*. Hypermethylation with Chromatin Status

**Figure 1:**
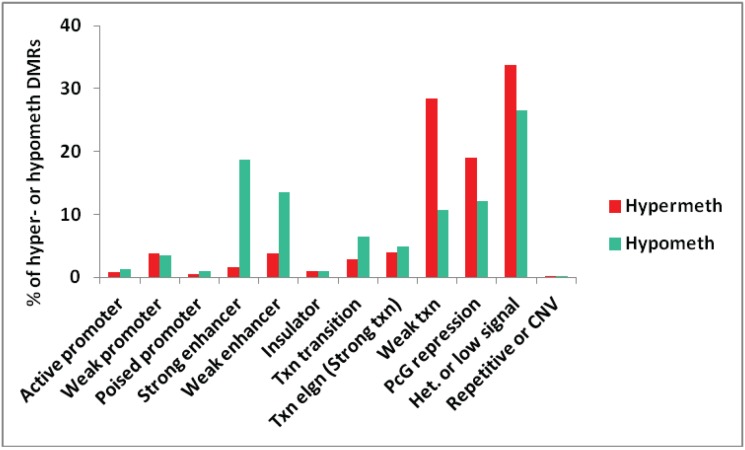
Genome-wide assessment of the type of chromatin segments overlapping MbMt-hypermethylated or hypomethylated DMRs. The percentages of all MbMt-hypermethylated DMRs and of all MbMt-hypomethylated DMRs (using the stringent criteria described in Methods) that overlap a given chromatin state [28] in Mb are shown. Active, weak, or poised promoter, chromatin enriched in H3K4me3; enhancer, chromatin containing H3K4me1 and H3K27Ac; PcG repression, chromatin marked by H3K27me3; Txn elgn, transcription elongation-type chromatin (also called Strong txn) enriched in H3K36me3; Het. or low signal, H3K9me3-type heterochromatin or low signal for H3K27Ac, H3K27me3, H3K4 methylation, H3K36me3, H3K79me2, H3K20me1, and CTCF binding; CNV, copy number variation.

We also tested for genome-wide associations between all the myogenic hypermethylated or hypomethylated DMRs and chromatin epigenetics in Mb as characterized in ENCODE profiles of chromatin state segmentation by HMM ([29], Bradley Bernstein, Broad Institute). The chromatin state analysis depends on whole-genome profiles obtained by ChIP-seq for standard histone modifications and for CTCF binding indicative of insulator function. An algorithm was then employed to deduce the type of chromatin in segments along the genome for the analyzed cell types [37]. For the comparison in Figure 1, we used more stringent selection criteria than in subsequent figures, requiring DMRs to cover at least three sites, have a mean PMD of at least ±0.3, and a log *p*-value < −12. We also removed from the analysis all DMRs that overlapped more than one type of chromatin state, resulting in a subset of 679 hypermethylated and 1391 hypomethylated DMRs. 

Overall, the distributions of chromatin states associated with hypermethylated and hypomethylated DMRs were significantly different (*p* < 0.001, Chi-squared test). As expected, many MbMt-hypomethylated DMRs were associated with strong enhancer-type chromatin (enriched in H3K4 monomethylation, H3K4me1, and H3K27acetylation, H3K27ac) while there was very little overlap of MbMt-hypermethylated DMRs with strong enhancer chromatin (*p* < 0.0001, Chi-squared test for difference of proportions, adjusted for multiple comparisons using the Bonferroni correction). The hypomethylation/strong enhancer associations were among the strongest seen for myogenic DMRs with chromatin state. There was also a strong association of the hypomethylated DMRs with weak enhancers (enriched in H3K4me1 but with little H3K27ac [28]) relative to hypermethylated DMRs (adjusted *p* < 0.0001). Although the hypermethylated DMR/weak enhancer overlap was modest, it was much more than the hypermethylated DMR/strong enhancer overlap. This suggests that DNA methylation can inhibit H3K27 acetylation to help downmodulate enhancer activity without abolishing it. With respect to promoter-type chromatin, the numbers of MbMt-hypermethylated DMRs and MbMt-hypomethylated DMRs overlapping active promoter chromatin (enriched in H3K4me3 and H3K27ac) were both very low (Figure 1).

Some studies have described negative or positive associations of DNA methylation with Polycomb group-type histone H3 lysine 27 trimethylation (H3K27me3), which is usually repressive [38,39]. We found both hypermethylated and hypomethylated MbMt DMRs often overlapped Polycomb-associated chromatin (PcG repression) although the percentage of hypermethylated DMRs in PcG repression chromatin was a little higher (adjusted *p* = 0.0005). DNA methylation can be antagonistic to H3K27me3 [38] but in some chromatin segments these two epigenetic parameters colocalize [39]. Whether low or high levels of methylation are associated with H3K27me3 probably depends on the context of the gene, the cell type, and the nature of surrounding chromatin. 

Transcription-transition chromatin (enriched in H3K36me3, H3K20me1, and H3K4me1; [28]) was significantly more frequently associated with hypomethylated *vs*. hypermethylated DMRs (adjusted *p* = 0.01). There was no significant difference in the association of transcription-elongation chromatin (enriched in H3K36me3 [28]) with hypermethylated *vs*. hypomethylated DMRs. This was unexpected because H3K36me3 in genome-wide studies has been found to be positively associated with DNA methylation [12]. 

Some specific gene regions with myogenic DMRs illustrating these complex relationships between DNA methylation, chromatin epigenetics, and gene expression are described below, as summarized in Table 1. For some of these gene regions, DNA methylation correlates with silencing of transcription (*MARVELD2*) but, for other regions, it does not, even when the methylation is in the extended promoter region (*TBX15*). Although DNA hypomethylation was much more frequently associated with enhancer-type chromatin (e.g., *TEAD4* and *LSP1*) than was DNA hypermethylation (Figure 1), there was no strong association of types of genes with hypomethylated DMRs at enhancer-type chromatin (H3K4me1 and H3K27Ac enrichment) and gene ontology (GO) designations for molecular function as determined by GREAT analysis [40]. For hypermethylated DMRs overlapping repressed-type chromatin (H3K27me3 enrichment) or transcribed-type chromatin (H3K36me3 enrichment), there was highly significant enrichment for the following GO molecular function term, sequence-specific DNA binding (FDR Q-values for site hits 8E-48 and 2E-17, respectively). Hypomethylated DMRs overlapping either repressed-type or transcribed-type chromatin did not exhibit a strong association with specific GO terms. Detailed analyses of four representative genes’ epigenetics and expression is consistent with the complex, context-dependent relationships between epigenetic changes and gene expression changes, although some of the expected relationships were seen in the analyses of both the whole genome and representative genes (e.g., the above-mentioned enhancer-type chromatin/DNA hypomethylation association). 

**Table 1:** Associations between myogenic differentially methylated regions (DMRs) and overlapping chromatin state for four genes ^a^.

Location of Myogenic Differential Methylation	MbMt DMR	Muscle DMR	Gene Product	Previous Relationship to Muscle?	Txn in Mb	Type of Chromatin at the Mb DMR	Figure (Region #)
**Hypermethylated DMRs**							
MARVELD2 , promoter, exon 1, intron 1 chr5:68710817-68711681	yes	yes	epithelial membrane protein	no	little or none	txn elongn	Figure 2, Figure S1 & Figure S2
TBX15 intron 1	yes	yes	T-box txn factor; directs devel. fates	only from txn	strong	weak txn	Figure 4 (1)
chr1:119522311-119522976							
TBX15 intron 1	yes	no				txn elongn	Figure 4 (2)
chr1:119526030-119527882							
TBX15 Mb promoter	yes	no				low signal	Figure 4 (3)
chr1:119531075-119533079							
TBX15 upstream	yes	yes				low signal	Figure 4 (4)
chr1:119535724-119537175							
TBX15 upstream	yes	yes				weak txn & weak enhancer	Figure 4 (5)
chr1:119542040-119543970							
TBX15 upstream	yes	yes				weak enhancer	Figure 4 (6)
chr1:119544699-119544773							
TBX15 upstream	yes	yes				PcG-repressed	Figure 4 (7)
chr1:119549291-119551164							
**Hypomethylated DMRs**							
TEAD4 intron2	no	yes	txn factor binding the M-CAT element in promoters or enhancer of muscle genes	yes	strong	weak enhancer	Figure 5 (1)
chr12:3073292-3073324							
TEAD4 intron2	no	yes				txn transition	Figure 5 (2)
chr12:3082851-3082995							
TEAD4 5' end of intron 3	yes	no				strong enhancer	Figure S6 (3)
chr12:3108155-3108180							
TEAD4 3' end of intron 3	yes	yes				txn elongn	Figure 7 (4)
chr12:3119130-3119880							
TEAD4 intron 5	yes	yes				strong enhancer	Figure 6 (5)
chr12:3123399-3123468							
TEAD4 exon 7	1 DM site	yes				txn elongn	Figure S7 (6)
chr12:3127664-3127741							
TEAD4 intron 10	yes	yes				strong enhancer	Figure S5 (7)
chr12:3141923-3142819							
LSP1 lymphoid extended promoter, exon 1, intron 1 (muscle only)	no	yes	F-actin binding	no	strong	weak enhancer & weak txn	Figure 8 (1)
chr11:1875260-1876267							
LSP1 Mb extended promoter	yes	yes				weak promoter, strong promoter & strong enhancer	Figure 8 (2)
chr11:1888801-1896175							
LSP1 last intron	yes	yes				strong enhancer	Figure 8 (3)
chr11:1912289-1912705							

^a^ Chromatin states are from ENCODE/Chromatin state segmentation analysis. Txn, transcription; devel., developmental. The regions overlapping low signal had the designation in the ENCODE database of heterochromatin (H3K9me3) or low signal but inspection of the ENCODE/Histone modifications dataset revealed that they did not contain H3K9me3 or H3K27me3 so they are identified as “low signal” chromatin segments in this table.

### 3.2. MARVELD2 0.3 kb Upstream of Constitutively Expressed RAD17: Myogenic Hypermethylated DMR at a Promoter Strongly Repressed in Myoblasts but without Repressive Histone Modifications

**Figure 2:**
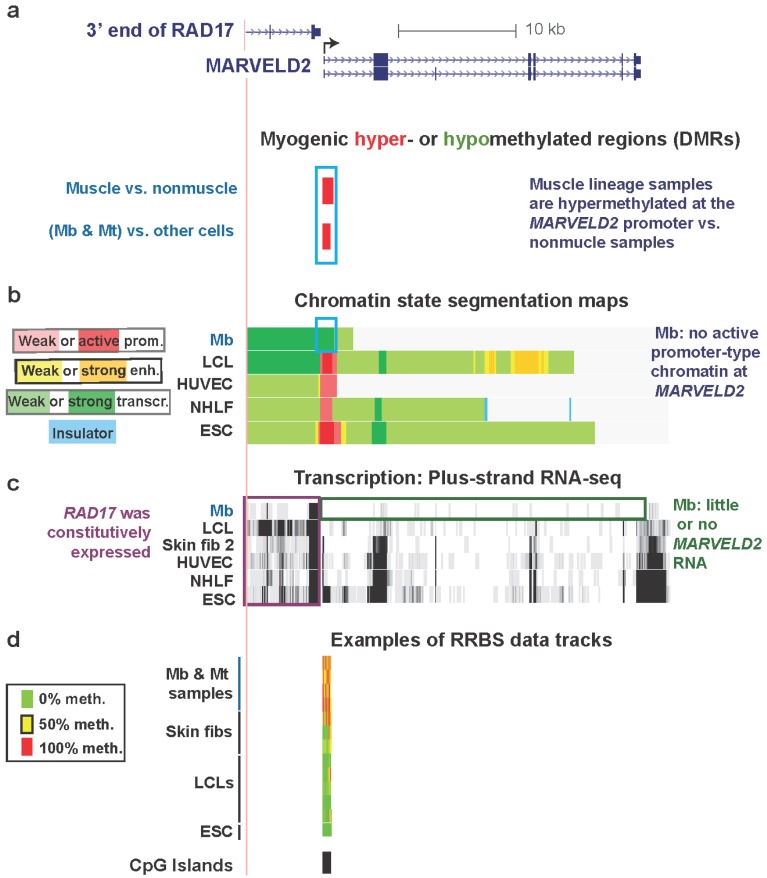
Myogenic hypermethylation at the silenced promoter of *MARVELD2* without repressive chromatin marks. (**a**) RefSeq genes in this region (chr5:68704307-68740247, hg19) are shown. Only one of nine *RAD17* isoforms is illustrated because all isoforms are the same in the 3' region that is depicted. Significantly hypermethylated (red) or hypomethylated (green, none seen) DMRs for the set of (Mb + Mt) samples *vs*. 16 types of nonmuscle cell cultures and for skeletal muscle *vs*. 14 types of nonmuscle tissue are aligned under the RefSeq genes. (**b**) The predicted chromatin structure (enh, enhancer; prom, promoter; transcr, transcriptionally active-type) based mostly on histone modifications [28]. (**c**) RNA-seq data for the plus-strand (vertical viewing range, 1-to-10). In this region, no specific signal was seen for the minus-strand. (**d**) Examples of RRBS data and the positions of CGIs. Using an 11-color, semi-continuous scale (see color guide), the RRBS tracks indicate the average DNA methylation levels at each monitored CpG site from the quantitative sequencing data [9,18]. The RRBS data are shown for four independent Mb cultures and Mt preparations from them; one female skin and two foreskin fibroblast cultures; five independent lymphoblastoid cell lines (LCLs), and H1 embryonic stem cells (ESC). The blue, purple, and green boxes indicate noteworthy features. In this and subsequent figures, all tracks are aligned, are from the UCSC Genome Browser [29], and are ENCODE data except for the differential methylation tracks and the MyoD binding tracks in subsequent figures, which are custom tracks.

Promoter regions are usually constitutively unmethylated, especially if they overlap CGI, even in genes with cell type-specific expression [2]. Nonetheless, there are notable exceptions in untransformed cell types as illustrated in Figure 2 for *MARVELD2*. This is a gene that has myogenesis-associated DNA hypermethylation at the promoter region and myogenesis-associated replacement of active promoter-type chromatin, seen in other cell types, with transcription-elongation chromatin in Mb (Table 1). It encodes a membrane protein that is found at tight junctions in epithelial cells and is also involved in normal hearing [41]. *MARVELD2* is strongly repressed in Mb and Mt (Figure 2c and Table S1, ENCODE/Strand-specific RNA-seq, long RNA-seq, >200 nt poly(A)^+^ RNA, Tom Gingeras, Cold Spring Harbor Laboratory, and ENCODE/RNA-seq, >200 nt poly(A)^+^ RNA, Barbara Wold, California Institute of Technology) and displays a hypermethylated DMR in Mb and Mt spanning from immediately upstream to downstream of the TSS (Figure 2a,d). 

Because 5-hydroxymethylcytosine (5hmC) can be mistaken for 5-methylcytosine (5mC) by RRBS, we used an enzymatic assay to ensure that we are correctly interpreting RRBS signal as 5mC. This assay quantifies 5mC and 5hmC at a given CCGG site. In several types of DNA samples, we examined the CCGG site 406 bp downstream of the *MARVELD2* TSS and residing within the myogenic DMR. The assay involves glucosylation of 5hmC by T4 phage β-glucosyltransferase (β-GT; Epimark, New England Biolabs), digestion with MspI or HpaII, and real-time PCR [18]. The results confirmed that Mb contained much higher amounts of 5mC at the examined site than did skin fibroblasts (average of three samples each, 95 and 35%, respectively, with <0.01% detectable 5hmC). In addition, skeletal muscle was hypermethylated relative to blood and brain (averages of two to three samples each, 54% 5mC and 5% 5hmC for skeletal muscle; 6% 5mC and 11% 5hmC for cerebellum; and 6% 5mC and 8% 5hmC for leukocytes). This finding of no detectable 5hmC at the assayed DMR site in the examined cultured cells and only low amounts in tissues is not surprising because levels of 5hmC are usually very much lower than those of 5mC in human DNA [42], and we previously showed that Mb had yet lower levels of this base than did skeletal muscle tissue [18]. 

Unlike Mb, a number of other cell types that were analyzed as part of the ENCODE project [27] do express *MARVELD2* (Figure 2c and Table S1). Among the 18 types of cell cultures studied, high levels of CpG methylation overlapping the CGI at the promoter region were observed only in Mb and Mt and one of the three skin fibroblast lines (Figure S1e, Skin fib 1). Skin fib 1 came from a 10-year-old girl as opposed to Skin fib 2 and Skin fib 3, both of which were from neonatal foreskin. The different epigenetics of Skin fib 1 *vs*. the other two samples could reflect the previous finding that skin fibroblasts in very different positions in the body can have different DNA epigenetics [43]. The nine myogenic progenitor samples were derived from muscle biopsies of four control individuals (Mb3, Mb7, Mt3, and Mt7), a patient with inclusion body myositis (IBM), and four patients with facioscapulohumeral muscular dystrophy (FSH). Although the control Mb and Mt samples appeared somewhat more hypermethylated than the FSH samples (Figure S1e) there was not a statistically significant difference between them and the normal Mb and Mt samples. In general, FSH and control Mb and Mt were similar in their DNA methylation profiles [18]. 

Gene-repressive DNA hypermethylation in promoter regions can localize to chromatin with repressive histone modifications, H3K9me3 or H3K27me3 [5,44]. However, this was not the case for the promoter region of *MARVELD2* (Figure S2). Instead, in Mb this TSS-overlapping DMR resided in transcription-elongation chromatin (Figure 2b). A similar histone modification pattern was observed in Mt (Figure S2c). In contrast, active promoter chromatin at the same region was seen in a lymphoblastoid cell line (LCL), human umbilical vein endothelial cells (HUVEC), normal human lung fibroblasts (NHLF), human mammary epithelial cells (HMEC), and embryonic stem cells (ESC) (Figure 2 and Figure S2; ENCODE Histone modifications and Chromatin state segmentation by HMM, [29], Bradley Bernstein, Broad Institute [28]). In addition, at this hypermethylated DMR, DNaseI hypersensitivity (ENCODE, Open Chromatin/Duke University, [18,45]) was suppressed in Mb and Mt *vs*. HUVEC, ESC, HMEC, normal human epidermal keratinocytes (NHEK), fetal lung fibroblasts (IMR-90), and osteoblasts (Figure S1c and data not shown). We do not have expression or histone modification data for skin fibroblast 1 but the sharply reduced DHS peak at the 5' end of *MARVELD2* in these cells (Figure S1c) makes it likely that, as for Mb and Mt, it has little or no expression of this gene in association with its promoter hypermethylation.

Key to understanding the hypermethylation in Mb, Mt, and skin fibroblast 1 is probably the close proximity to the 5' end of *MARVELD2* of the nearest upstream gene, *RAD17*, a cell cycle checkpoint gene. The 3' end of *RAD17* is only 0.3 kb (Figure 2a) from the TSS of *MARVELD2.* *RAD17* is expressed in all examined cell types, including Mb (Figure 2c). Considering that the 3' ends of RefSeq genes represent the 3' terminus of the corresponding mRNA just upstream of the poly(A) tail, which is added after endonucleolytic cleavage of the nascent transcript, the actual ends of the transcripts from *RAD17* are likely to overlap the TSS of the downstream *MARVELD2*. Because *MARVELD2* is an epithelial membrane-associated gene, it probably requires tight repression in the muscle lineage, whose differentiation and repair involve muscle-lineage-specific cell-cell interactions and fusion. The myogenic *MARVELD2* hypermethylation is probably functioning like extensive methylation of other upstream promoter regions and regions immediately downstream of the TSS to usually repress gene expression, as determined by genome-wide studies [9] and experimental systems [46,47,48,49].

In the promoter region of this gene in Mb and Mt, the presence of transcription-elongation chromatin, rather than standard repression-associated chromatin marks, further suggests that a repressive role for DNA methylation here is separate from typical promoter-inhibiting chromatin epigenetics. We hypothesize that the use of a large hypermethylated DMR for tissue-specific repression at the *MARVELD2* promoter allows silencing of *MARVELD2* in Mb and Mt while avoiding interference with generation of the 3' end of the *RAD17* transcript. The maintenance of this promoter hypermethylation in skeletal muscle (Figure 2a) makes it likely that DNA-methylation-mediated repression of this gene is necessary in the skeletal muscle lineage at the tissue stage as well as at the myogenic progenitor stage, especially because retention of MbMt-hypomethylated sites in muscle is very unusual [18], as described above.

### 3.3. TBX15: Myogenic Hypermethylation Surrounding the 5' End of a Gene that Is Moderately Expressed in Myoblasts

**Figure 3:**
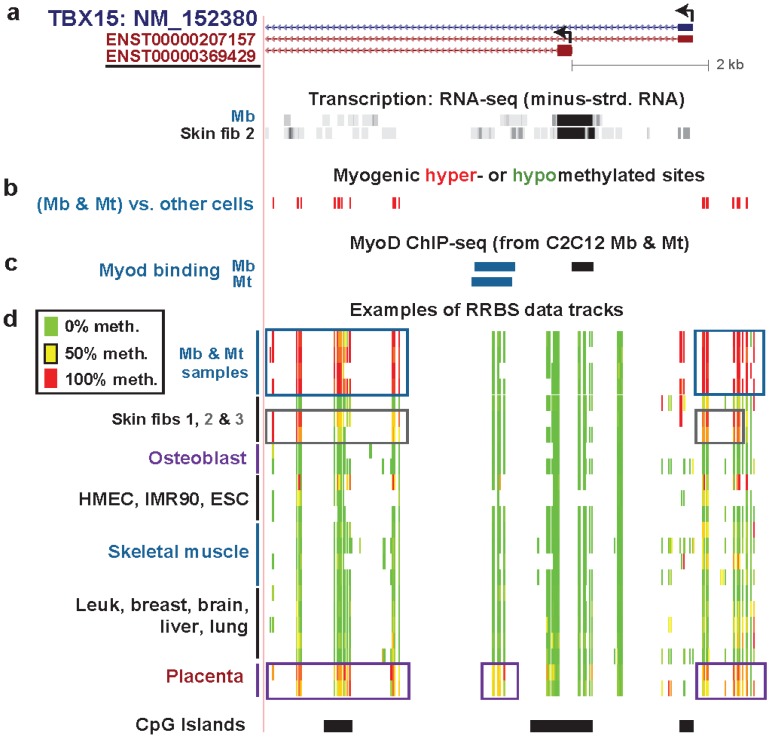
*TBX15*, preferentially expressed in myogenic cells: Myogenic hypermethylation immediately upstream and downstream of the empirically observed TSS. (**a**) Minus-strand RNA-seq data for Mb and foreskin fibroblasts are shown aligned under the single RefSeq gene isoform and two ENSEMBL isoforms. The underlined ENSEMBL isoform most closely resembles the observed RNA-seq signal from the minus-strand in Mb and foreskin fibroblasts. In this region (chr1:119525932-119533279), no specific signal was seen for the plus-strand (vertical viewing range, 1-to-10). The much lower expression of *TBX15* in HMEC, ESC, HUVEC, NHEK, NHLF, and LCL cell cultures is shown in Figure S3 and Table S1. Very high expression in the osteoblast sample (Ost) is indicated by the H3K79me2 signal shown in Figure S4. (**b**) MbMt-hypermethylated CpG sites (red) or hypomethylated sites (green, none). There were no muscle-hypermethylated or hypomethylated sites in this region. (**c**) MyoD binding from C2C12 ChIP-seq [54] and identification of orthologous human sequences. Sites shown in blue overlapped variants of the MyoD consensus sequence (CAGCTG, V$MYOD_01, V$MYOD_Q6, or E47 sites from Conserved TFBS [29]). (**d**) Examples of RRBS data and the positions of CGIs as in Figure 2. Technical duplicates are shown for the osteoblast, placenta, and two different skeletal muscle samples. The gray box indicates the hypermethylation in the two foreskin fibroblast samples (Skin fibs 2 and 3) that was not present in the fibroblasts from the skin of a 10-year-old girl (Skin fib 1). Placental hypermethylated sites and MbMt-hypermethylated sites are also indicated with boxes.

**Figure 4:**
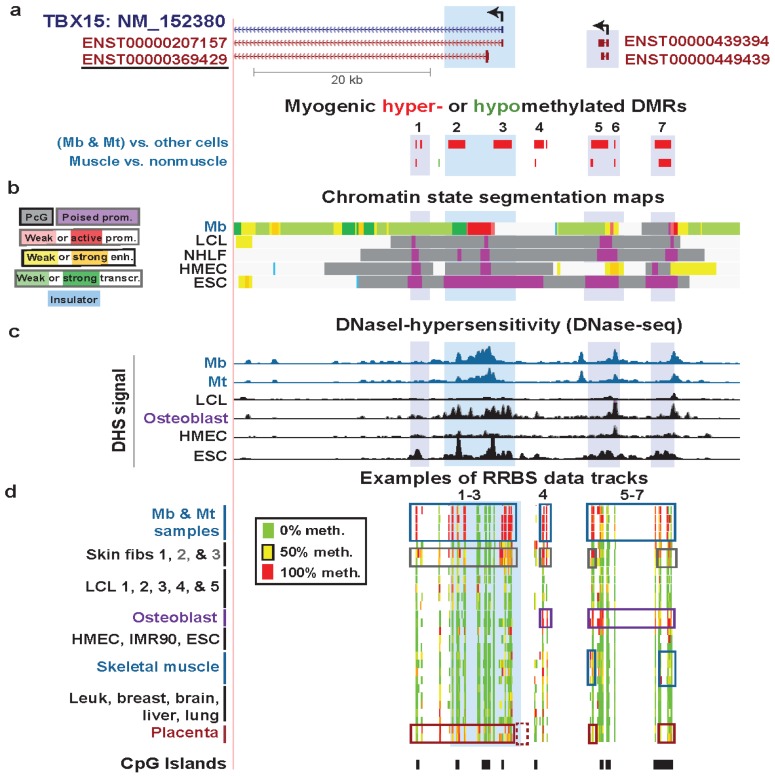
Association of hypermethylated myogenic DMRs with boundaries of active promoter-like chromatin in *TBX15* and further upstream. (**a**) The underlined ENSEMBL isoform is the one that the RNA-seq data supports for Mb and Skin fibroblast 2 (foreskin fibroblasts; Figure 3). MbMt-hypermethylated DMRs (red) and one hypomethylated DMR (green) are indicated. The hypermethylated DMRs or clusters of DMRs are numbered for reference in the text. (**b**) Chromatin state segmentation analysis as in Figure 2. (**c**) DNaseI hypersensitivity signal (ENCODE/Open chromatin by DNaseI HS/Duke University [17,32]). (**d**) Examples of RRBS data tracks for the indicated samples as in Figure 3 but for a larger region (chr1:119501896-119558791). The dotted box at the bottom shows a region previously studied for DNA methylation in placenta by Chelbi *et al*. [55], as described in the text.

*TBX15* encodes a T-box transcription factor that regulates many developmental pathways, including mesoderm specification, adipocyte differentiation, and chondrocyte differentiation [50,51]. Little is known about its role in the skeletal muscle lineage but the gene was reported to be highly expressed in skeletal muscle tissue [52]. In addition, its expression is a marker common to skeletal muscle progenitors and brown adipocytes [53]. Immediately adjacent to the TSS of the single RefSeq gene isoform (NM_152380), there was a cluster of hypermethylated MbMt DM sites (Figure 3b) that are part of one of many DMRs surrounding the TSS (Figure 4a). In Mb, these DMRs reside in chromatin domains with the following character by chromatin state segmentation analysis [28]: transcription-elongation, weak enhancer, weak transcription, low-signal (low histone modifications), and PcG-repression (Table 1). MbMt DMRs most frequently overlapped the latter three chromatin states in the genome-wide analysis (Figure 1).

Despite the myogenic hypermethylation close to and upstream from its 5' end, *TBX15*, unlike *MARVELD2*, was moderately expressed in Mb (Figure 3). Because we set a higher threshold for identifying individual DM sites than for each DM site within a DMR, and precision is needed when characterizing how close DNA methylation is to the TSS, we refer to DM sites rather than DMRs in the following discussion. The location of the 5' end of the gene, as expressed in Mb, is important for understanding this gene’s DNA methylation profile. The 5' end of the RefSeq NM_152380 *TBX15* isoform differs from the end deduced from strand-specific RNA-seq data for Mb and foreskin fibroblasts (Figure 3a, ENCODE, strand-specific RNA-seq, poly(A)^+^ RNA, Cold Spring Harbor Laboratory) and also for chondrocytes, osteoblasts, and undifferentiated mesenchymal stem cells (ENCODE data not shown, strand-specific RNA-seq, total RNA, Cold Spring Harbor Laboratory). It is closer to that of the ENSEMBL ENST00000369429 isoform than to the RefSeq isoform and ~1.6 kb from the 5' end of the RefSeq isoform (Figure 3a).

Moreover, the position of the active promoter-type chromatin seen specifically in Mb (Figure 4b), which overlaps a constitutively unmethylated DNA region, is consistent with the use of the first exon in ENST00000369429 rather than that of NM_152380. Cufflinks analysis of RNA-seq data indicated considerable amounts of ENST00000369429 RNA in Mb and foreskin fibroblasts while there was none detected in HUVEC, NHEK, NHLF, LCL, and ESC samples (Table S1A). Preferential expression of *TBX15* in myogenic progenitor cells (Table S1B) is consistent with the finding that near the 5' end of this gene (Figure 3c) there are DNA sequences that are orthologous to C2C12 mouse Mb and Mt sequences bound by the myogenic transcription factor MyoD in ChIP-seq [54]. 

The myogenic hypermethylated sites are only ~150 bp from the TSS of the *TBX15* RefSeq isoform but ~1.8 kb from the first exon of ENST00000369429. Because extensive regions of DNA methylation very close to a TSS are implicated in transcription suppression [3,46], one function of this cluster of strongly hypermethylated sites might be suppressing the use of the RefSeq TSS in Mb, Mt, and foreskin fibroblasts. Osteoblasts might use differential availability of specific transcription factors to direct promoter usage rather than epigenetics because they exhibited the same predominant TSS as seen in Mb and skin fibroblasts (see above) despite the lack of DNA methylation at the RefSeq TSS in these cells.

The question remains as to why *TBX15* has many additional hypermethylated DM sites (and DMRs) in Mb and Mt extending from ~8 kb downstream of the deduced TSS to ~21 kb upstream (Figure 4) despite its substantial expression in Mb and Mt. Insights from further analysis of epigenetic and expression data suggest some explanations. The profiles for DNaseI hypersensitivity, histone H3K4me3, and H3K27ac show that around the gene’s 5' end there was little or no open chromatin and active promoter-like chromatin in cell types that were not expressing this gene (LCL, HMEC, NHLF, and ESC) while there was open and active-promoter-like chromatin (Mb and foreskin fibroblasts) for cell types expressing this gene (Figure 4b,c; Figure S4c, H3K4me3, H3K4me2, and H3K27ac). Osteoblasts express this osteogenesis-associated gene [56] even more strongly than Mb and foreskin fibroblasts, as can be deduced from their large enrichment for H3K79me2 at the 5' end of this gene and their H3K36me3 signal [57] further downstream of the 5' end (Figure S4). H3K79me2 is associated with transcriptional activity and is enriched in the region between the initiation marks, H3K4me3 or H3K4me2, and the elongation mark K36me3 [58,59]. Skeletal muscle and placenta also preferentially express *TBX15* [52,60]. Osteoblasts, skeletal muscle, and placenta displayed much more DNA methylation than most other samples in Regions 1–3, 4, and/or 5–7 surrounding the 5' end of *TBX15* although methylation in these regions was more extensive in Mb and Mt (Figure 3d and Figure 4d). However, although osteoblasts had strong methylation of the upstream Regions 4–7, they lacked methylation in Regions 1–3 close to the RNA-seq-determined TSS. 

We propose that methylation in Mb, Mt, and foreskin fibroblasts at Regions 1–3, which includes sequences 1.6 kb upstream of the RNA-seq-determined TSS, is responsible for the lower, although still considerable, expression of this gene in these cells compared to that in osteoblasts. Methylation in these regions frames the ~2.6-kb active promoter region in Mb (Figure 4b), which might prevent it from spreading to the wider, ~11.6 kb active promoter region seen in osteoblasts (Figure S4). This methylation may thereby down-modulate the Mb and foreskin fibroblast promoter activity to a moderate level. In contrast, we propose that at further upstream Regions 4–7 (Figure 4a,d), DNA methylation helps upregulate transcription by counteracting putative repressive elements or by serving as boundaries around nearby ncRNA genes. There are a couple of overlapping ncRNA genes in the vicinity of *TBX15*-upstream hypermethylated DMRs in a region with enhancer-like and weak promoter-like chromatin in Mb (Figure 4a,b), which the hypermethylation might help control. 

Interestingly, Chelbi *et al*. [55] saw a small, but highly significant, decrease in DNA methylation in the immediate-upstream region of *TBX15* in pathological placentas (vascular intra-uterine growth restriction, vIUGR; Figure 4d, dotted box at bottom). This is a *TBX15*-upstream subregion where we do not have RRBS data, and it was the only subregion investigated in the vIUGR study. DNA hypomethylation in this subregion in vIUGR placentas was significantly associated with worse symptoms and, surprisingly, with less expression of the gene [55]. These findings are consistent with the hypothesis that Region 4 (Figure 4a) and the adjacent region studied by Chelbi *et al*. contains *cis*-acting transcription down-regulatory sequences, whose transcriptional repression is averted by DNA methylation, possibly by preventing binding of repressor proteins [55]. 

*TBX1*, another T-box gene family member is implicated in the symptoms of the DiGeorge syndrome [61]. Like *TBX15*, *TBX1* is preferentially expressed in Mb and Mt and displays MbMt hypermethylation in the 5' region [18]. *TBX1* exhibits even more myogenic hypermethylation in its 3' region. While haploinsufficiency for *TBX15* is linked to the DiGeorge syndrome, which affects many parts of the body including the heart, loss of most or all TBX15 protein results in the Cousin syndrome, involving bone deformities [62]. Both *TBX15* and *TBX1* probably have to be tightly regulated in a cell type-specific manner to prevent pathological consequences, and both may be using cell type-specific DNA methylation to help achieve this fine-tuning of expression. 

### 3.4. TEAD4: Many Myogenic Hypomethylated DMRs in the Body of a Gene that Is Highly Transcribed in Myoblasts

**Figure 5:**
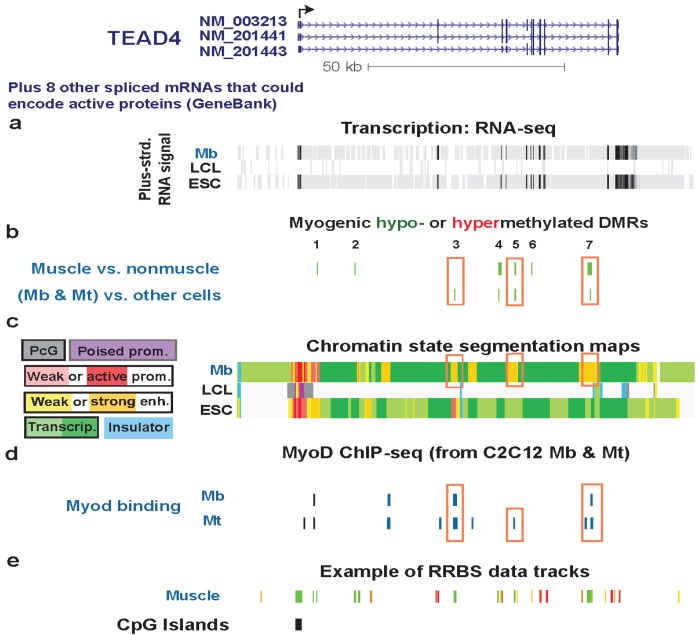
Multiple myogenic hypomethylated DMRs in the gene body of *TEAD4*. (**a**) RNA-seq data are shown from the plus-strand as in Figure 2 except that the vertical viewing range was 1-to-10. (**b**) Myogenic hypomethylated DMRs are indicated (green). There were no hypermethylated DMRs. (**c**) Chromatin state segmentation analysis is illustrated as in Figure 2. The orange boxes in this track set mark strong enhancer-type chromatin that overlapped myogenic DMRs. (**d**) As in Figure 3c, human sequences orthologous to mouse sequences binding MyoD in C2C12 Mb or Mt are shown. Orange boxes in this track indicate inferred MyoD binding sites that overlap MbMt or muscle DMRs. (**e**) One track of skeletal muscle RRBS data is included to show the RRBS coverage in this chr12:3053101-3169038 region.

Like *TBX15*, *TEAD4* encodes a transcription factor that is preferentially, but not exclusively, expressed in the skeletal muscle lineage, as determined from a study of mice [63]. *TEAD4* also displayed myogenic differential methylation although, in this case, the intragenic DMRs were hypomethylated rather than hypermethylated (Figure 5b), and three of them resided in strong enhancer-type chromatin specifically in myogenic progenitor cells (Figure 5c and Table 1). The other DMRs overlapped chromatin with the histone modifications of weak enhancers, transcription elongation regions, or transcription transition regions (transitioning from promoter-type to transcription-type chromatin) [28]. This gene was more highly expressed in Mb and especially in Mt than in HUVEC, NHEK, NHLF, LCL, and ESC samples, as determined by our Cufflinks analysis of non-strand-specific RNA-seq profiles derived from poly(A)^+^ RNA (Table S1). In Mb and Mt, the high level of expression of this gene is consistent with its upregulating expression of the important myogenic transcription factor gene *MYOG* during Mb differentiation to Mt [64]. 

**Figure 6:**
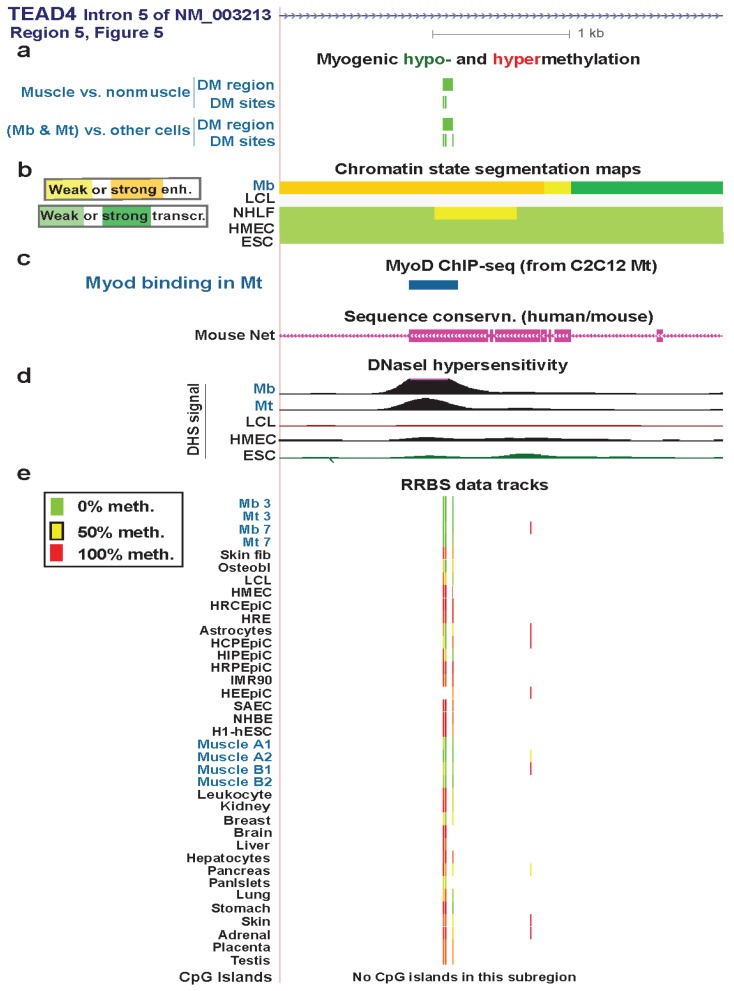
A myogenic hypomethylated DMR in *TEAD4* that overlaps Mb-associated enhancer-type chromatin. (**a**) Only the NM_003213 transcript is shown here because the other two transcripts (Figure 5) are indistinguishable in this subregion. Both myogenic DMRs and DM sites are shown. Only hypomethylation (green) was seen in this region. (**b**) Chromatin state segmentation as in previous figures. (**c**) Inferred MyoD binding sites as in Figure 3. There was a MyoD binding sites in this subregion in C2C12 Mt but not in Mb. The UCSC Genome Browser track for human/mouse sequence conservation [29] is shown. (**d**) and (**e**) DNaseI hypersensitivity and RRBS tracks are depicted as in previous figures; some of the biological replicates and most technical duplicates are omitted. The region shown is chr12:3122206-3125443.

The three *TEAD4* hypomethylated MbMt DMRs residing in strong enhancer-type chromatin in Mb (Regions 3, 5, and 7, Figure 5) did not overlap enhancer-type chromatin in LCL, NHLF, HMEC, or ESC samples. Their myogenesis association was further evidenced by their location overlapping or next to intronic DNA sequences orthologous to MyoD binding sites in C2C12 myogenic progenitor cells (Figure 6, Figure S5, and Figure S6). Two of these DMRs were adjacent to peaks of DNaseI hypersensitivity specific to Mb and Mt *vs*. the other examined cell types (Figure 6 and Figure S5). Therefore, these DMRs are likely to be part of myogenesis-associated enhancers. That skeletal muscle tissue displayed hypomethylated DMRs in two of these three Mb enhancer-like regions (Figure 6 and Figure S5) suggests the persistence of enhancer activity at the muscle stage. This could contribute to the higher expression of *TEAD4* in skeletal muscle than in other human tissue types [65]. 

Regions 1 and 2 were DMRs in skeletal muscle tissue and not in myogenic progenitor cells (Figure 5b,c). In Mb, they overlapped weak enhancer-type chromatin and transcription-transition-type chromatin, respectively. These regions might become strong enhancers only in skeletal muscle tissue and not at the progenitor stage.

**Figure 7:**
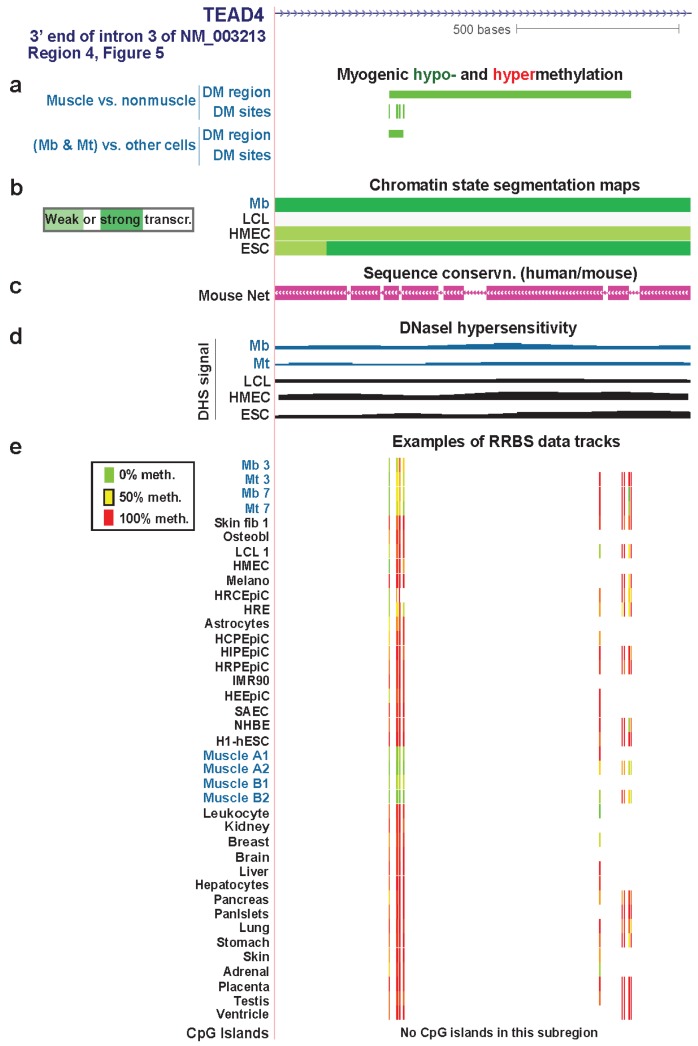
A myogenic hypomethylated DMR in *TEAD4* that overlaps transcription-elongation type chromatin. (**a**), (**b**), (**c**), (**d**) and (**e**) Myogenic hypo- and hypermethylation, Chromatin state segmentation, sequence conservation, DNaseI-hypersensitivity, and RRBS data tracks are shown as in Figure 6. We illustrate RRBS data for heart ventricle in this figure that was not available for the region in Figure 6. However, ventricle data, unlike RRBS data shown for the other tissues, was not used in determination of skeletal muscle differential methylation. The region shown is chr12:3118782-3120061.

The intronic myogenic DMRs at Regions 4 and 6 (Figure 5) were embedded in chromatin exhibiting histone modifications indicative of transcription elongation (Figure 7 and Figure S7). In Mb and Mt, Region 4 was a DMR but Region 6 had only a single DM site. In skeletal muscle tissue, these regions were DMRs containing more DM sites than in Mb and Mt. This suggests spreading of DNA hypomethylation in *cis* over time or with differentiation to skeletal fibers. These regions were not associated with peaks of transcription-activating chromatin epigenetic marks (H3K4 methylation, H3K27ac, or H3K20me1) or DNaseI hypersensitivity. However, they did contain mouse/human conserved sequences (Figure 7c and Figure S7 and data not shown). 

Region 4 is in intron 3 of the main Mb-expressed isoform of *TEAD4* (NM_003213) in Mb and Mt. Region 6 is in intron 6 but extended into exon 7 in skeletal muscle tissue (Figure S7). Hypomethylation in Region 6 might participate in regulating the efficiency of differential splicing because the upstream exon was an alternative exon. Given that there are mRNA structures supporting eleven, and not just three, RNA variants (AceView, [66]), it is possible that Region 4 also affects co-transcriptional processing of the primary transcript. Alternatively, these MbMt hypomethylated sites might represent early stages in forming a myogenic enhancer or some other transcription-promoting *cis*-acting structure.

Whatever the role of hypomethylation at Regions 4 and 6, the lack of the expected open-chromatin epigenetic marks at sites with Mb and Mt hypomethylation and the increase in hypomethylation in these regions in skeletal muscle suggest that these DNA methylation changes preceded chromatin epigenetic changes. Moreover, the greater extent of hypomethylation in these regions and at Regions 1, 2, and 7 in skeletal muscle than in myogenic progenitor cells suggests that *TEAD4* has a role at the tissue stage in the muscle lineage in addition to its demonstrated involvement, along with other TEAD factors, in controlling myogenesis [64]. In skeletal muscle tissue, its protein product might be important in the positive regulation of expression of genes responsible for the tissue-protective unfolded protein response [64].

### 3.5. LSP1: Hypomethylation at Tissue-Specific Promoter Chromatin in Myoblasts and Lymphoblasts

We previously briefly described the extraordinary specificity of MbMt- and muscle-hypomethylated DM sites *vs*. lymphoid hypomethylated sites at distant alternative promoters for *LSP1*, a gene named for its encoded lymphocyte-specific protein [10]. LSP1 is an intracellular protein that binds to F-actin and has been associated mostly with various leukocyte functions, including cell adhesion and migration [67]. Here we analyze for the first time *LSP1* chromatin state segmentation profiles and DMRs and also provide newly generated RNA-seq data for Mt as well as for Mb [16]. 

**Figure 8:**
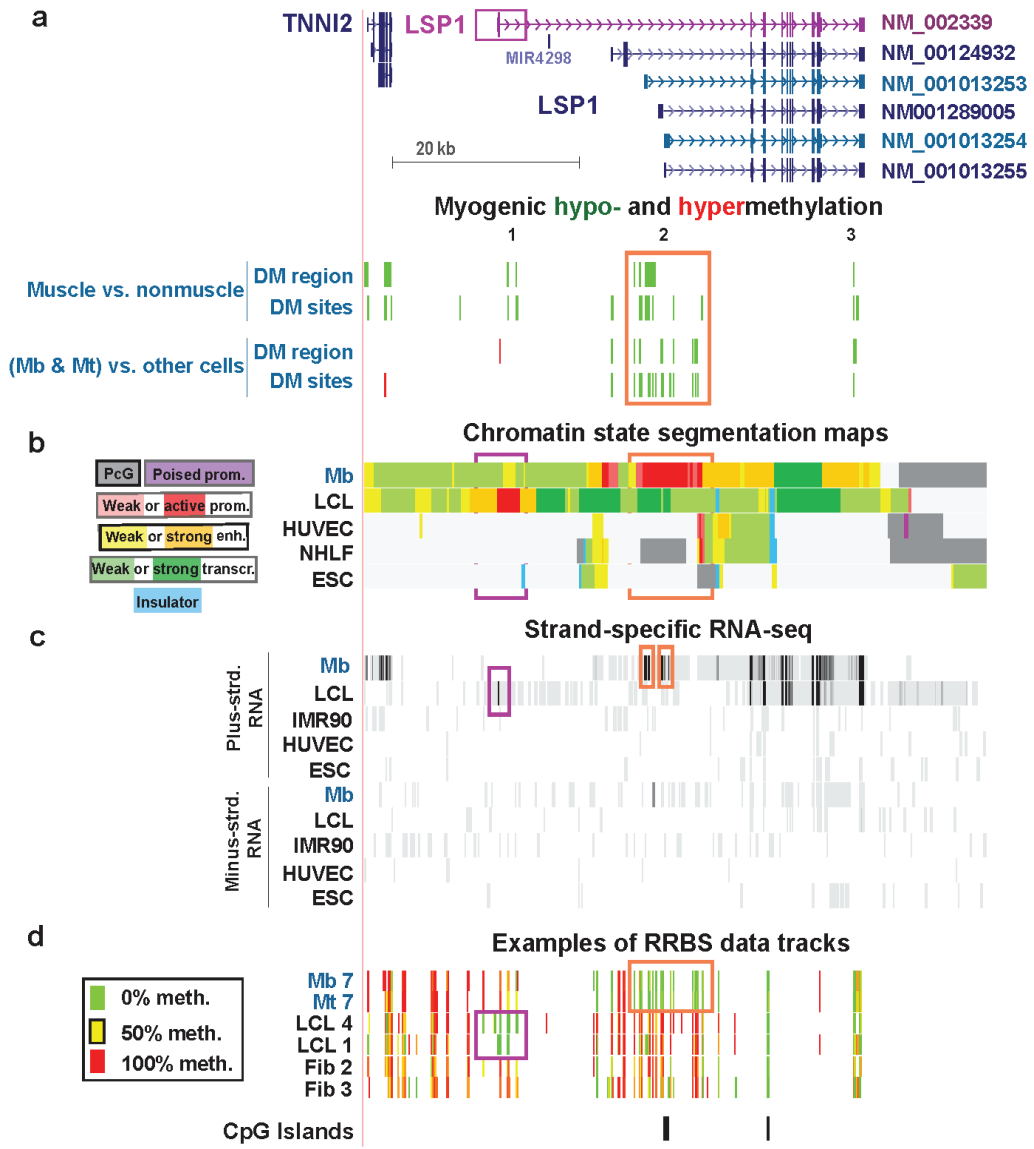
Myogenic hypomethylation *vs*. lymphoid hypomethylation at tissue-specific promoters of *LSP1*. (**a**) The main RefSeq isoforms detected in Mb and lymphoid cells are shown in blue-green and purple, respectively (chr11:1,859,948-1,926,584). Myogenic differential methylation is indicated as described for Figure 6. (**b**) Chromatin states are depicted as in previous figures. (**c**) Strand-specific RNA-seq data are shown for both strands with the vertical viewing range set at 1-to-100. The narrow orange rectangles indicate signal from alternative first exons of *LSP1* in Mb. The narrow purple rectangle marks the first LCL exon. In other tracks similar regions are boxed. Previously we concluded that the main *LSP1* transcript in Mb was ENST00000405957 [10], which is very similar to RefSeq transcript NM_001013253. However, new RNA-seq profiles (G. Crawford and M. Ehrlich labs [16]), which had greater depth of cDNA sequences for *LSP1* and used different Mb samples, indicate that NM_001013254 was the major *LSP1* transcript in three of four Mb or Mt samples and NM_001013253 was the major transcript in the fourth sample (Table S1); however, the multiplicity of *LSP1* isoforms complicates their resolution (see text). (**d**) Examples of RRBS data tracks for several of the myogenic progenitor cell, lymphoblastoid cell, and skin fibroblast samples.

Most of the MbMt DMRs and DM sites associated with *LSP1* were in a cluster that spans a long segment containing Mb-specific (and Mt-specific) active promoter-type chromatin (Figure 8a,b, orange boxes) [10]. Analysis of RNA-seq data for different RNA isoforms is complicated for this gene because there are 23 GeneBank mRNAs, including 12 probable alternative promoters and 21 alternatively spliced variants (AceView, [66]). Different RNA isoforms were seen in the LCL sample and in myogenic progenitor cells (Table S1). There are many different *LSP1* RNA isoforms and some encode variant polypeptides so that any influence of epigenetics on the relative amounts of RNA isoforms generated could change the relative amounts of the encoded proteins.

In the chromatin of Mb and Mt, the H3K79me2 signal (data not shown) began in the vicinity of the most distal Mb-specific exon in RNA-seq (Figure 8c, orange box on left), and the H3K79me2 signal in the LCL started near the far upstream, LCL-specific first exon (Figure 8c, purple box). This confirms the cell type-specific use of widely distanced promoter regions for Mb/Mt *vs*. lymphoid cells. Moreover, both Cufflinks analysis of ENCODE RNA-seq data for many cell types (not strand-specific, poly(A)^+^ RNA, Barbara Wold, California Institute of Technology and strand-specific RNA-seq, Tom Gingeras, Cold Spring Harbor Laboratory) indicate that there was no detectable transcription in Mb of the lymphoid-specific *LSP1* isoform (NM_002339/ENST00000311604). In an LCL, this was essentially the only RNA isoform detected and it was present at an extraordinarily high level (Table S1). The LCL-hypomethylated DM sites in the gene were far from the MbMt-hypomethylated DM sites and overlapped the LCL active-promoter chromatin in five different LCLs (Figure S8f). Leukocytes showed hypomethylation in part of the LCL promoter region, and skeletal muscle was hypomethylated in part of the Mb/Mt promoter region (Figure S8f). Therefore, although tissue-specific hypomethylation at promoters is unusual, *LSP1* exhibits such hypomethylation of its alternative tissue-specific promoter regions.

In the first intron of the LCL-specific *LSP1* isoform, far upstream of the Mb promoter, there were two muscle-hypomethylated DMRs that were not present in Mb and Mt (Figure 8, Region 1). Proximal to these DMRs is *TNNI2*, a gene encoding a fast-twitch skeletal muscle protein that is specifically expressed in skeletal muscle [65]. The muscle DMRs in this region (Figure S8) may help upregulate expression of *TNNI2* at the muscle tissue stage. It is curious to see skeletal muscle-specific hypomethylation distant from the Mb/Mt promoter and close to the LCL/lymphoid promoter. Presumably hypomethylation in muscle of LCL-specific *LSP1* intron 1 did not lead to inappropriate lymphoblast-type *LSP1*expression in muscle. 

Lastly, the 3' terminal intron of all the RefSeq isoforms of *LSP1* harbors a MbMt- and muscle-hypomethylated DMR (Figure 8, Region 3). The conservation of this hypomethylation in muscle as well as in Mb and Mt and its isolated location at the end of the gene suggest a specific biological role. Given that the multiplicity of *LSP1* RNA variants includes seven validated alternative polyadenylation sites (AceView, [66]), hypomethylation in the 3' region might help control 3' end formation of *LSP1* RNA in the muscle lineage. 

The role played by LSP1 protein, its variants, and its intracellular F-actin binding activity in myogenic progenitor cells and adult skeletal muscle tissue is unknown. It might be related to the control of the special shape of Mt and skeletal muscle fibers or to the ability of this protein to regulate adhesion to fibrinogen matrix proteins. Whatever these functions are in skeletal muscle, its tissue-specific expression and the intricate muscle lineage-specificity of the epigenetics of *LSP1* indicate that there is tight regulation of expression of *LSP1* isoforms in skeletal muscle and its progenitor cells.

## 4. Conclusions

Our genome-wide analysis of the overlap of myogenic hypomethylated or hypermethylated DMRs with different classes of chromatin (e.g., weak or strong enhancer- or promoter-type chromatin) suggests the variety of ways that DNA methylation can be used in a chromatin context- and cell type-dependent manner to help regulate transcription. In addition, examination in detail of the epigenetics and expression of representative genes in many different cell types supports the hypothesis that DNA methylation helps regulate gene expression in more ways than are usually appreciated. Providing in-depth analyses of the epigenetics of a given gene region from genome-wide profiles of DNA methylation, histone modification, and DNaseI hypersensitivity can lead not only to the design of cogent experiments to test the effects of epigenetics on gene regulation but also to new insights into previously unknown gene functions.

## Supplementary Files

Supplementary File 1 Supplementary Materials (PDF, 666 KB)
