# Supplementary material for: Myogenic Differential Methylation: Diverse Associations with Chromatin Structure
Source: Biology (Basel). 2014 Jun 19;3(2):426–51. doi: 10.3390/biology3020426 (PMC4085616; doi:10.3390/biology3020426)
Supplement: Supplementary File 1 — Supplementary Materials (PDF, 666 KB) [file biology-03-00426-s001.pdf]

Supplementary Material

Table S1. RNA-Seq analysis of genes studied for their myogenic DMRs.

A. RNA-Seq data for Mb and nonmuscle cell cultures.

Gene name	Mapped RNA isoform	FPKM [1]					
		Mb	HUVEC	NHEK	NHLF	LCL	ESC
MARVELD2	ENST00000325631	0	0.9	5.8	1.3	<0.000	1.8
TBX15	ENST00000369429	9.0	0	0	0	0	0
TEAD4	ENST00000359864	34.6	13.4	4.4	6.2	0.7	18.3
LSP1	ENST00000405957	80.4	0	0	0.2	0	0
LSP1	ENST00000406638	30.2	0	0.1	0.02	0.3	0
LSP1	ENST00000311604	0	0	0.19	0	272	0

[1] We determined the FPKM (Fragments Per Kilobase of transcript per Million mapped reads) from ENCODE data (http://genome.ucsc.edu; non-strand-specific RNA-seq, poly(A)$^+$ RNA, B. Wold, CalTech) using Cuffdiff as described in Methods. RNA-seq signals were mapped to ENSEMBL isoforms. The isoforms with the highest signals are shown. Mt are not included because they are not available in the ENCODE data base.

B. RNA-Seq data for comparison of Mb and Mt for different genes.

Gene name	Mapped RNA isoform	FPKM [1]			
		Mb4	Mb13	Mt4	Mt13
MARVELD2	NM_001038603	0.2	0.2	0.4	0.4
TBX15	NM_152380	13.2	10	22.6	20.1
TEAD4	NM_003213	20.5	11.6	50.1	46.5
LSP1	NM_001013253	0.4	11.4	0.5	0.3
LSP1	NM_001013254	3.9	10.9	1.4	2.0
LSP1	NM_002339	0	0	0	0

[1] We determined FPKM from previously unpublished RNA-seq data on poly(A)$^+$ RNA using Cufflinks as described in the main text (Crawford/Ehrlich laboratories). Observed transcripts were mapped to RefSeq transcripts.

Figure S1. Myoblasts (Mb), myotubes (Mt) and one skin fibroblast sample were CpG-hypermethylated and lacked a DHS peak at the *MARVELD2* TSS. Tracks from the UCSC genome browser and samples are described in the main text. Among DNA from cultured cells, only the sample from the skin fibroblast culture derived from a 10-year-old (Skin fib 1, purple box) was as highly methylated in this region as Mb and Mt. For Panel **c**, the four other LCLs not displayed in this panel but shown in Panel **e** also had a DHS peak in this region and several of them had much higher peaks than did LCL1.

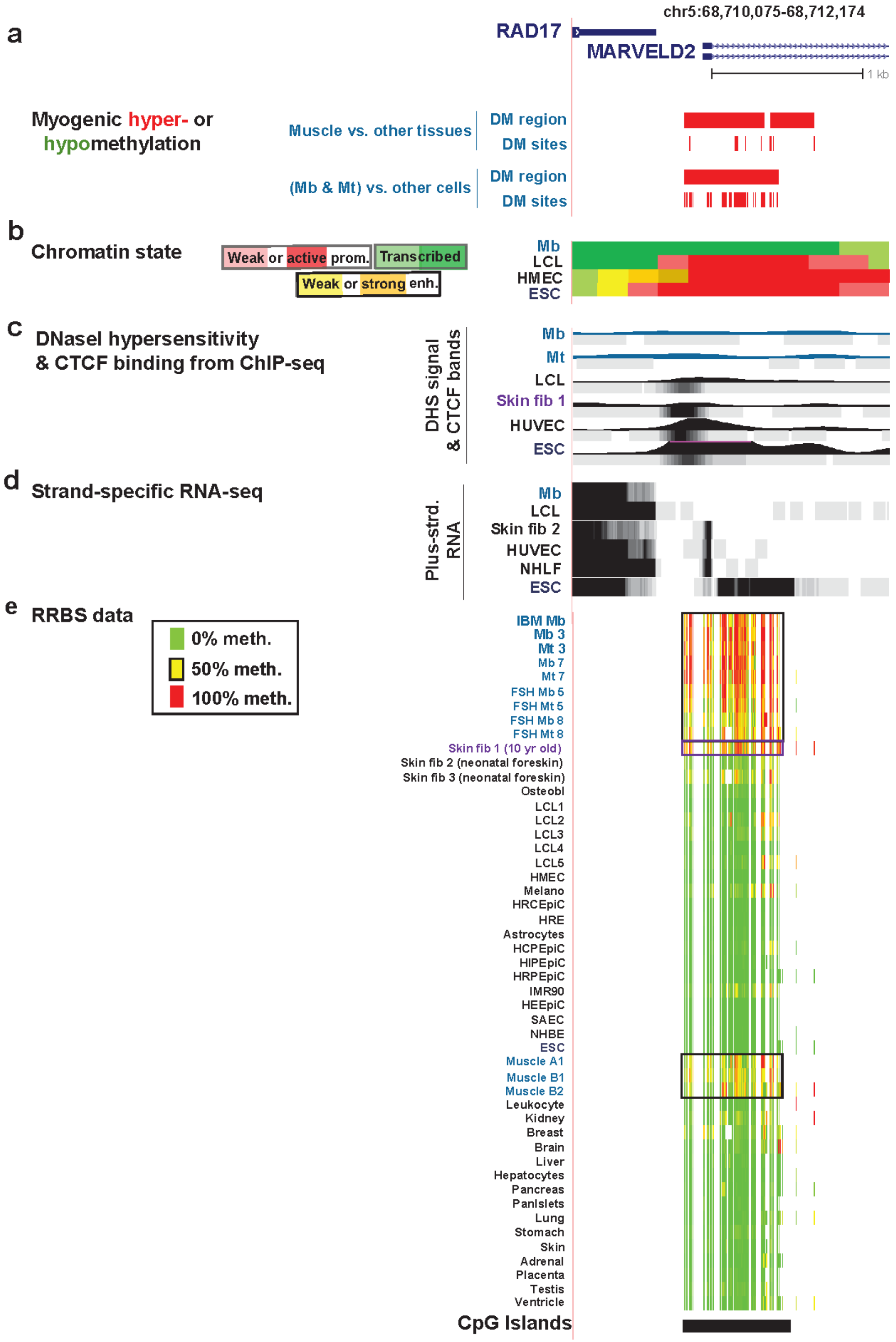

Figure S2. Lack of repression- or activation-associated histone modifications in Mb and Mt at the *MARVELD2* TSS. The RNA-seq track for non-strand-specific RNA-seq and histone modification tracks (ENCODE/Broad Institute, Bradley Bernstein) are shown for the indicated samples in the same region as in Figure S1.

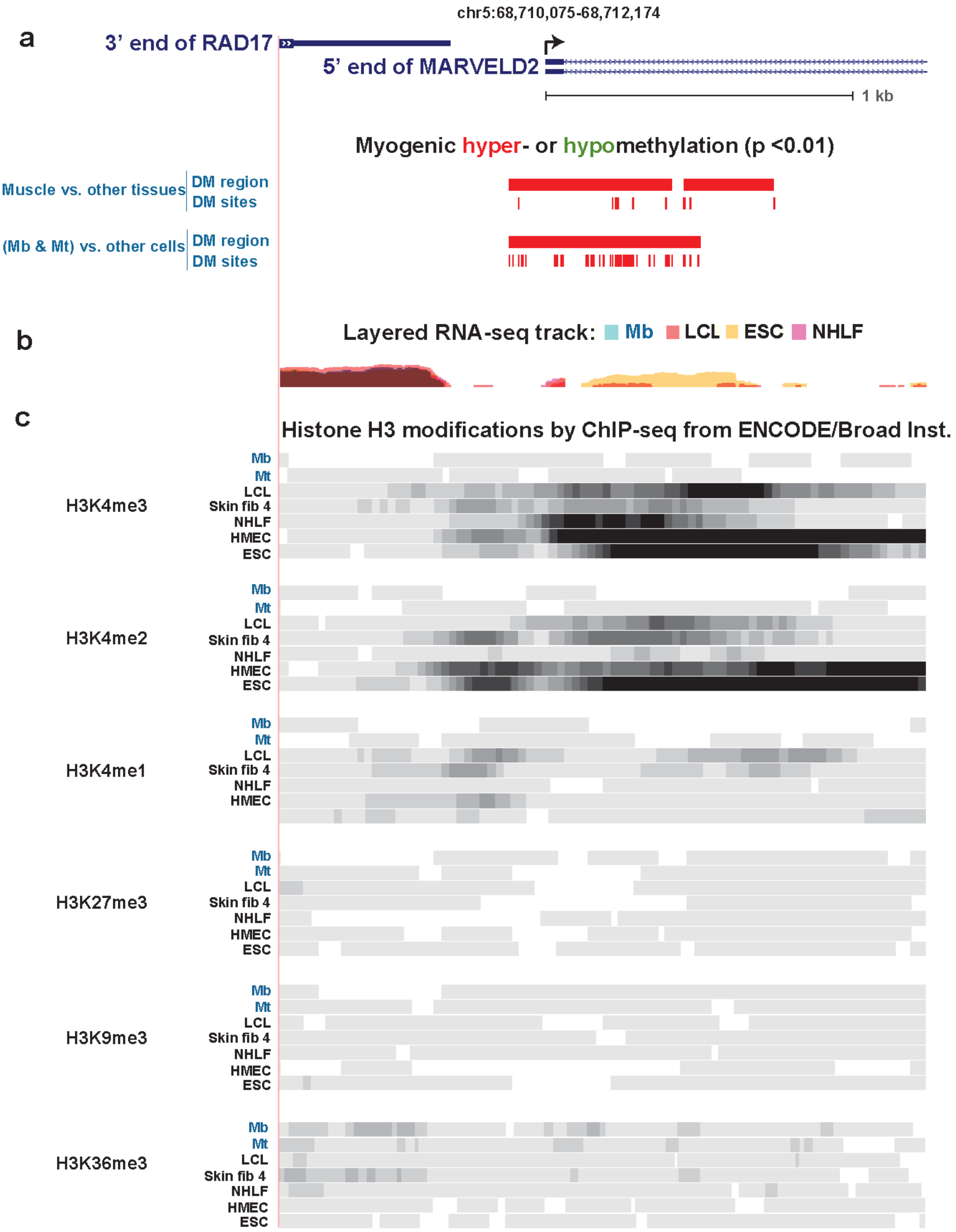

Figure S3. An active promoter region for *TBX15* was hypermethylated in its 5' vicinity. Tracks from the UCSC genome browser are shown as described in the main article. Note that the LCL RNA in the *TBX15* gene region came only from the 3' end of the gene. For reference, yellow highlighting indicates the entire transcribed region in the various tracks. The boxes in (g) show hypermethylation that correlates with transcription. Although some hypermethylation was seen in this region for HMEC, these cells did not transcribe this gene. This demonstrates, as expected, that this subregion's hypermethylation does not suffice for transcription.

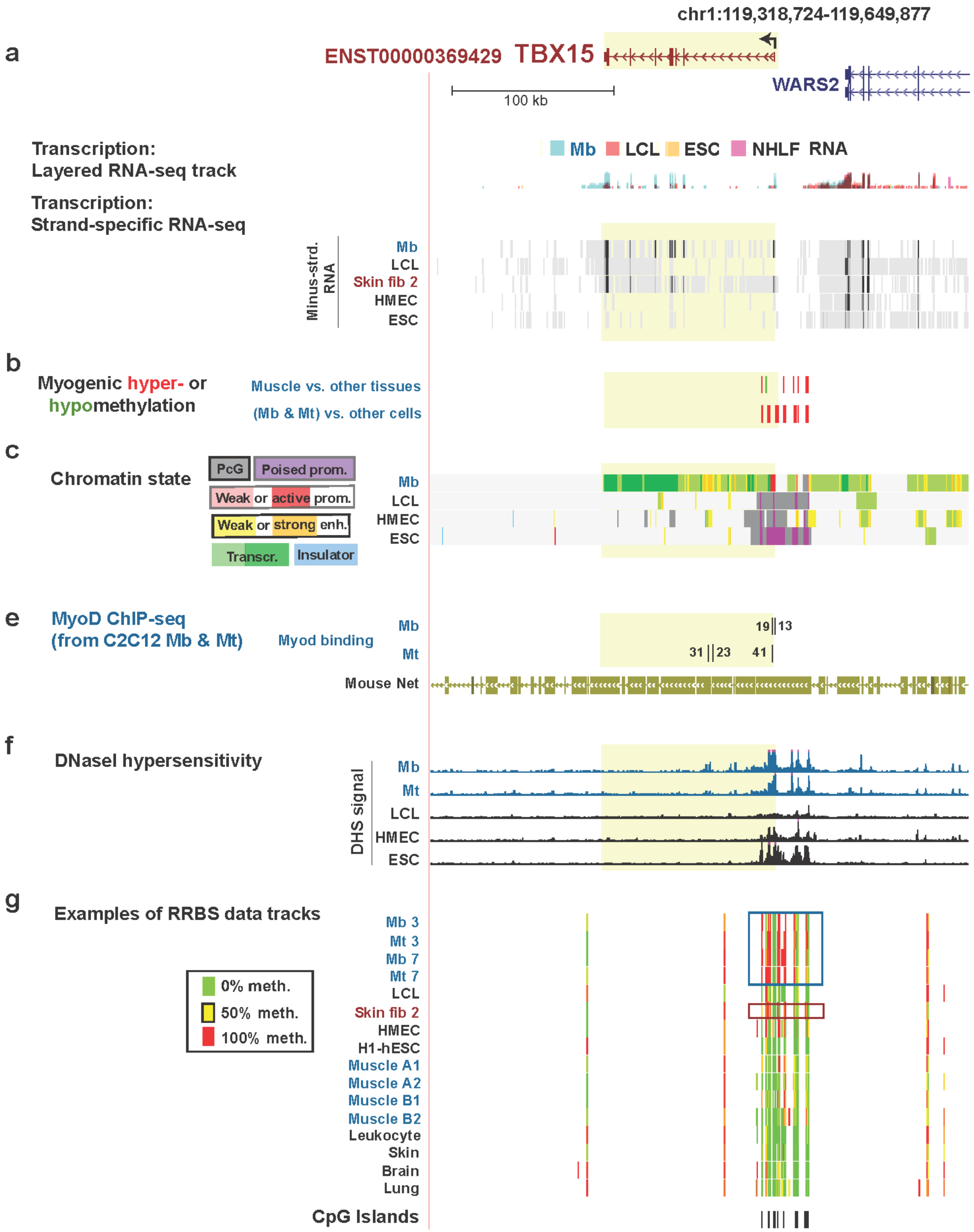

Figure S4. DNA methylation around the 5' end of *TBX15* is located at the borders of the wide osteoblast active-promoter region and at small promoter-like regions further upstream. Histone modification tracks are shown as in Figure S2 except that, in addition, CTCF binding and EZH2 binding from ChIP-seq (ENCODE/Broad Institute, Bradley Bernstein) are displayed. The yellow highlighted region was shown in Figure 3 of the main article. Light blue-highlighting indicates myogenic DNA hypermethylation that overlaps two ncRNA genes or H3K4methylation signal seen in osteoblasts, lung fibroblasts, mammary epithelial cells, or embryonic stem cells. No EZH2 data are available for osteoblasts. The region shown is the same as for Figure 4.

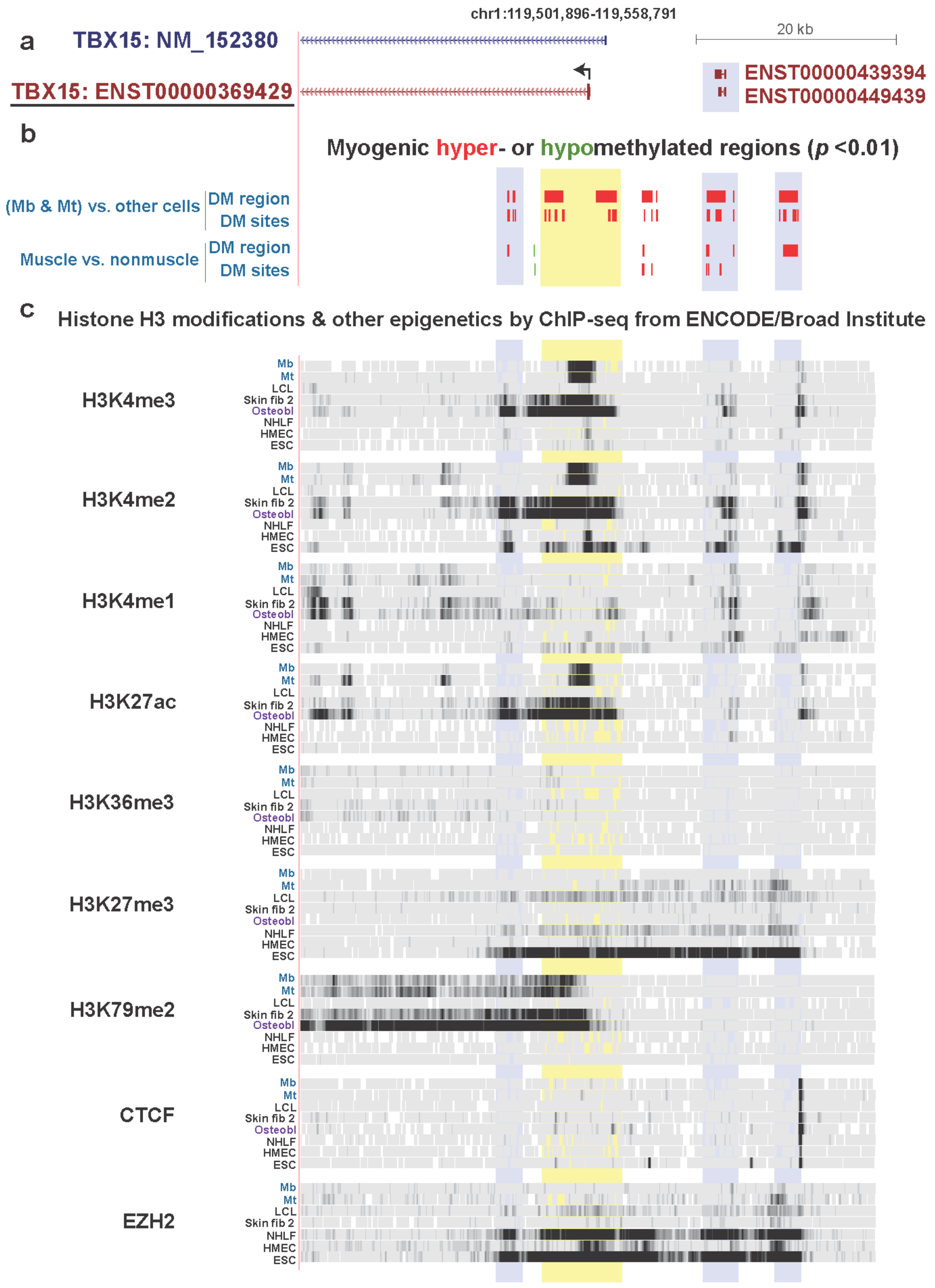

Figure S5. Myogenic hypomethylation, active enhancer chromatin, DNaseI hypersensitivity, and inferred MyoD binding in intron 10 of *TEAD4*. Tracks from the UCSC genome browser are shown as described in the main article.

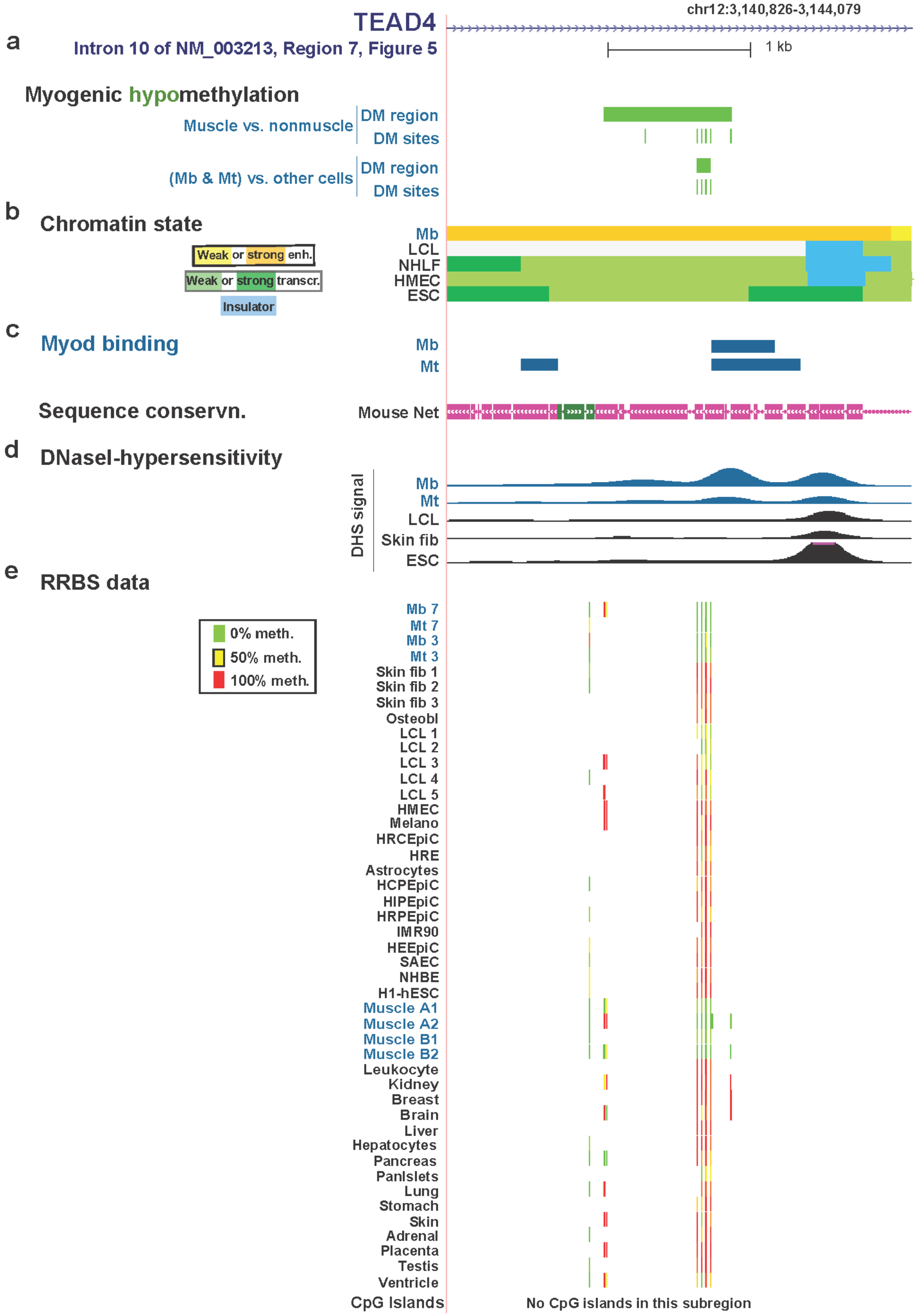

Figure S6. Myogenic hypomethylation, active enhancer chromatin, and inferred MyoD binding in a subregion of intron 3 of *TEAD4*. Tracks from the UCSC genome browser are shown as described in the main article except that here we include the RRBS tracks for facioscapulohumeral muscular dystrophy (FSH) Mb and Mt and inclusion body myositis (IBM) Mb. Mb3, Mt3, Mb7, and Mt 7 come from control individuals. For skeletal muscle, there was not enough of a difference between its low methylation level and the examined non-muscle samples for it to be significantly hypomethylated.

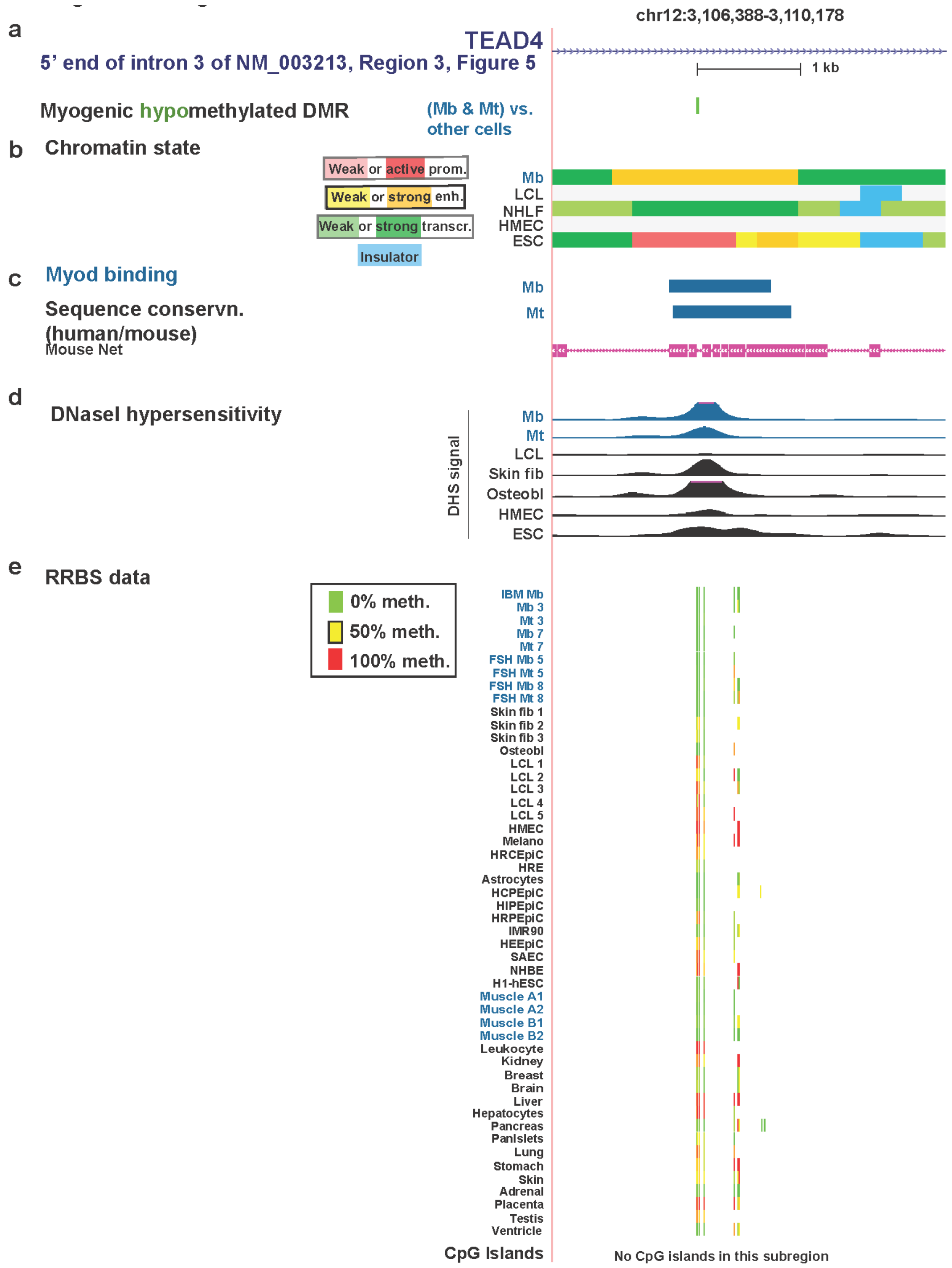

Figure S7. Myogenic hypomethylation around exon 7 of *TEAD4* in a region with histone modifications indicative of active transcription. Tracks from the UCSC genome browser are shown as described in Figure S6. In this figure, the three RefSeq isoforms are shown because of differences in exons in this region.

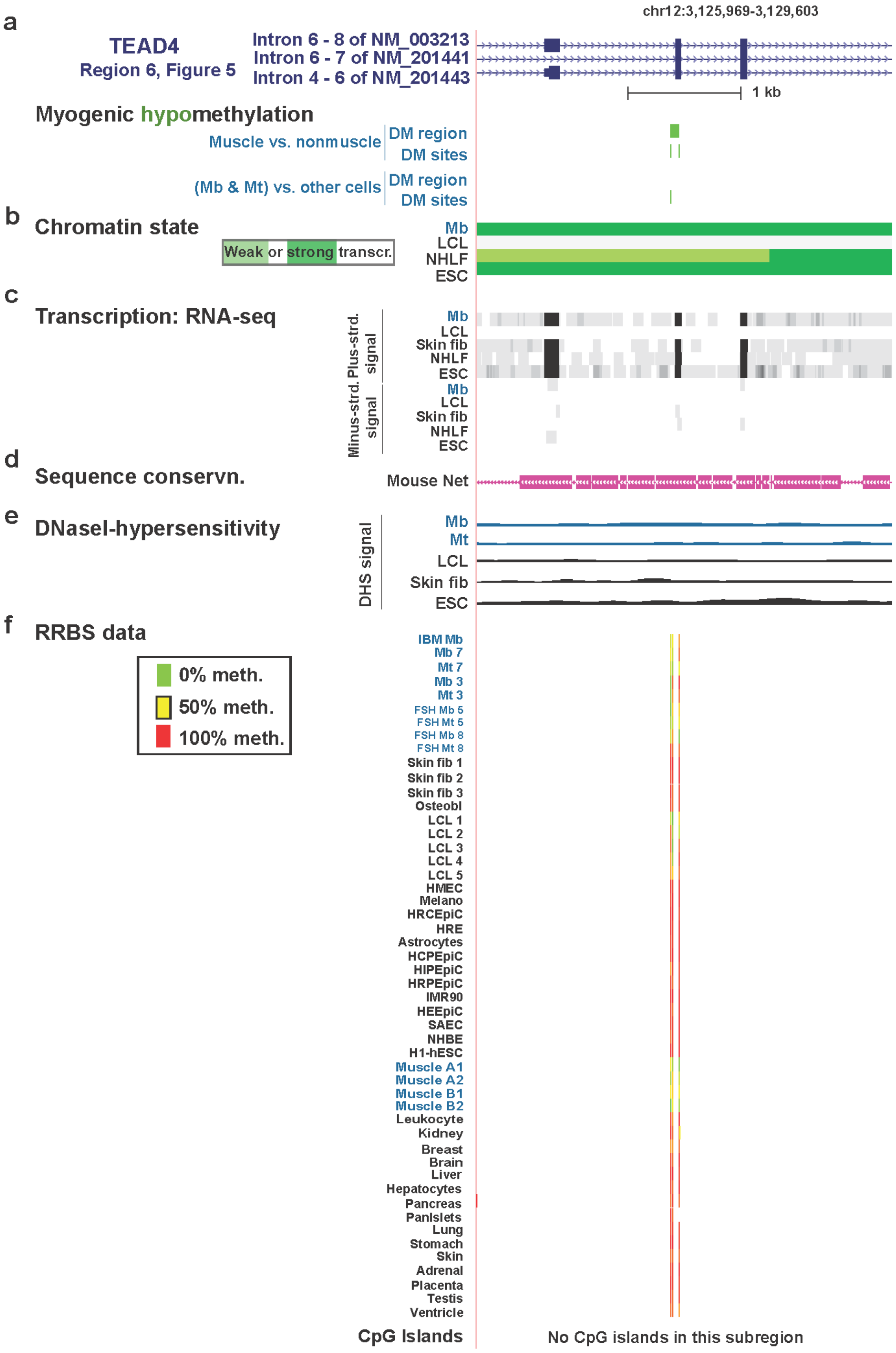

Figure S8. Expression of *LSP1* specifically in myogenic cells or lymphoblastoid cells was correlated with specific DNA hypomethylation. Tracks from the UCSC genome browser are described in the main text. The purple boxes denote lymphoid epigenetic features and the orange box shows the region of skeletal muscle hypomethylation.

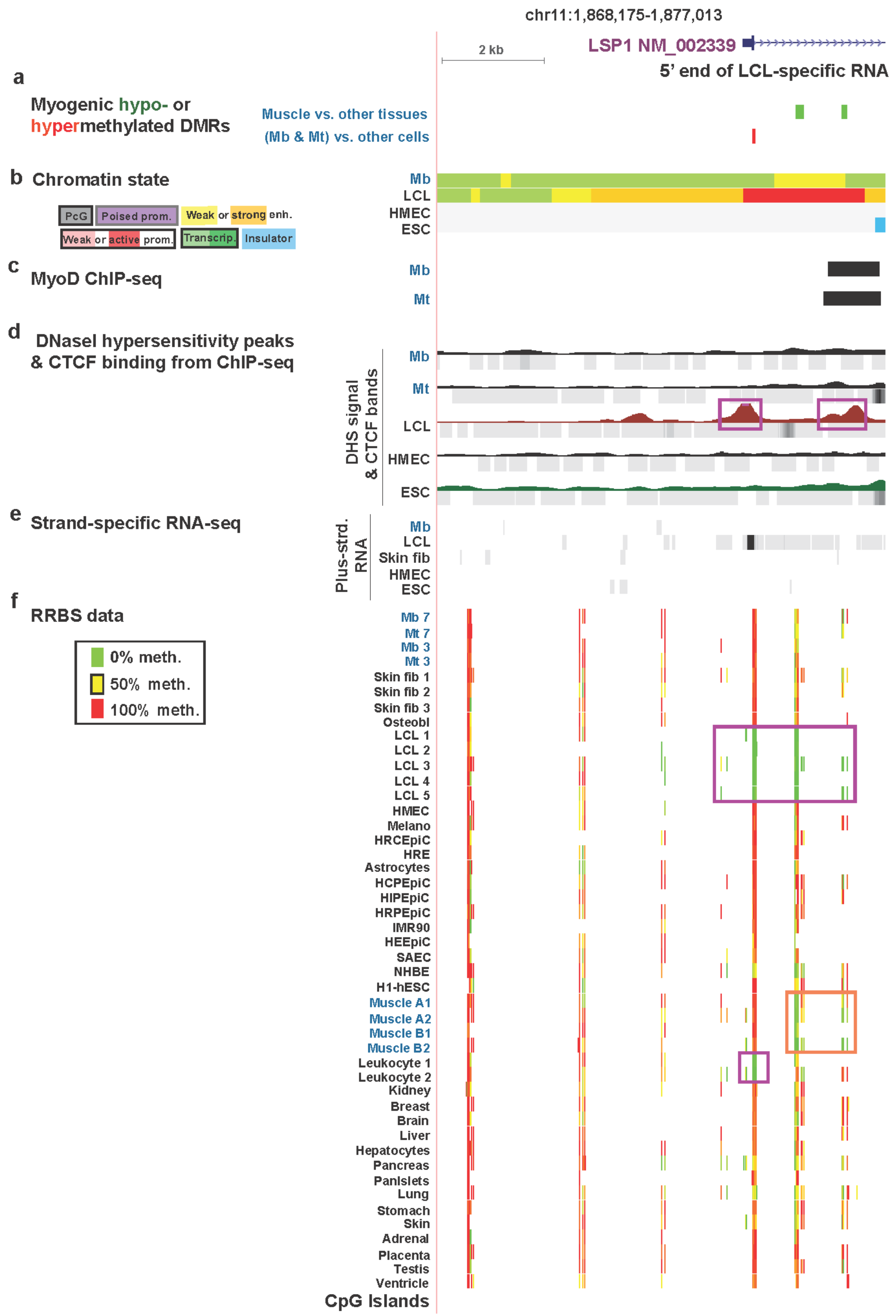

© 2014 by the authors; licensee MDPI, Basel, Switzerland. This article is an open access article distributed under the terms and conditions of the Creative Commons Attribution license (http://creativecommons.org/licenses/by/3.0/).
